# Arrestins: structural disorder creates rich functionality

**DOI:** 10.1007/s13238-017-0501-8

**Published:** 2018-02-16

**Authors:** Vsevolod V. Gurevich, Eugenia V. Gurevich, Vladimir N. Uversky

**Affiliations:** 1 Department of Pharmacology, Vanderbilt University, Nashville, TN 37232 USA; 2 Department of Molecular Medicine and USF Health Byrd Alzheimer’s Research Institute, Morsani College of Medicine, University of South Florida, Tampa, FL 33612 USA; 3 Institute for Biological Instrumentation, Russian Academy of Sciences, Pushchino, Moscow Region Russia 142290

**Keywords:** arrestin, GPCR, crystal structure, NMR, EPR, disorder, protein-protein interactions

## Abstract

Arrestins are soluble relatively small 44–46 kDa proteins that specifically bind hundreds of active phosphorylated GPCRs and dozens of non-receptor partners. There are binding partners that demonstrate preference for each of the known arrestin conformations: free, receptor-bound, and microtubule-bound. Recent evidence suggests that conformational flexibility in every functional state is the defining characteristic of arrestins. Flexibility, or plasticity, of proteins is often described as structural disorder, in contrast to the fixed conformational order observed in high-resolution crystal structures. However, protein-protein interactions often involve highly flexible elements that can assume many distinct conformations upon binding to different partners. Existing evidence suggests that arrestins are no exception to this rule: their flexibility is necessary for functional versatility. The data on arrestins and many other multi-functional proteins indicate that in many cases, “order” might be artificially imposed by highly non-physiological crystallization conditions and/or crystal packing forces. In contrast, conformational flexibility (and its extreme case, intrinsic disorder) is a more natural state of proteins, representing true biological order that underlies their physiologically relevant functions.

## Introduction

**Figure 1:**
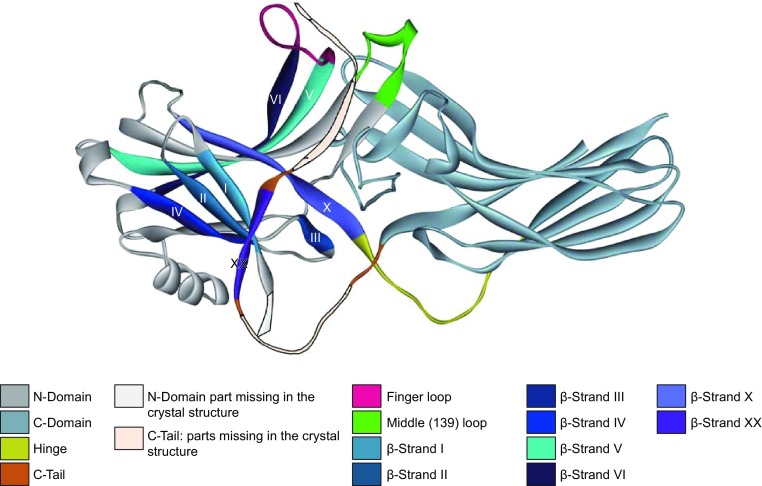
**Arrestins structure and structural elements**. The structure of arrestin-2 is shown, with main structural elements color-coded, as follows: N-domain, gray; C-domain, teal; inter-domain hinge, yellow; the part of the C-tail resolved in crystal structures, magenta; finger loop, pink; middle loop (termed 139-loop in arrestin-1), green. The part of the N-domain (the N-terminus) and of the C-tail that are not resolved in any crystal structure were added manually and are shown as light grey and light pink, respectively, with black outline. β-strands I, II, III, IV, V, VI, X, and XX are shown in indicated colors and labeled with roman numerals on the structure. Note that β-strand XX, anchored to β-strand I and -α-helix via three-element interaction (see Fig. 2A), includes virtually the whole part of the C-tail resolved in crystal structures

**Figure 2:**
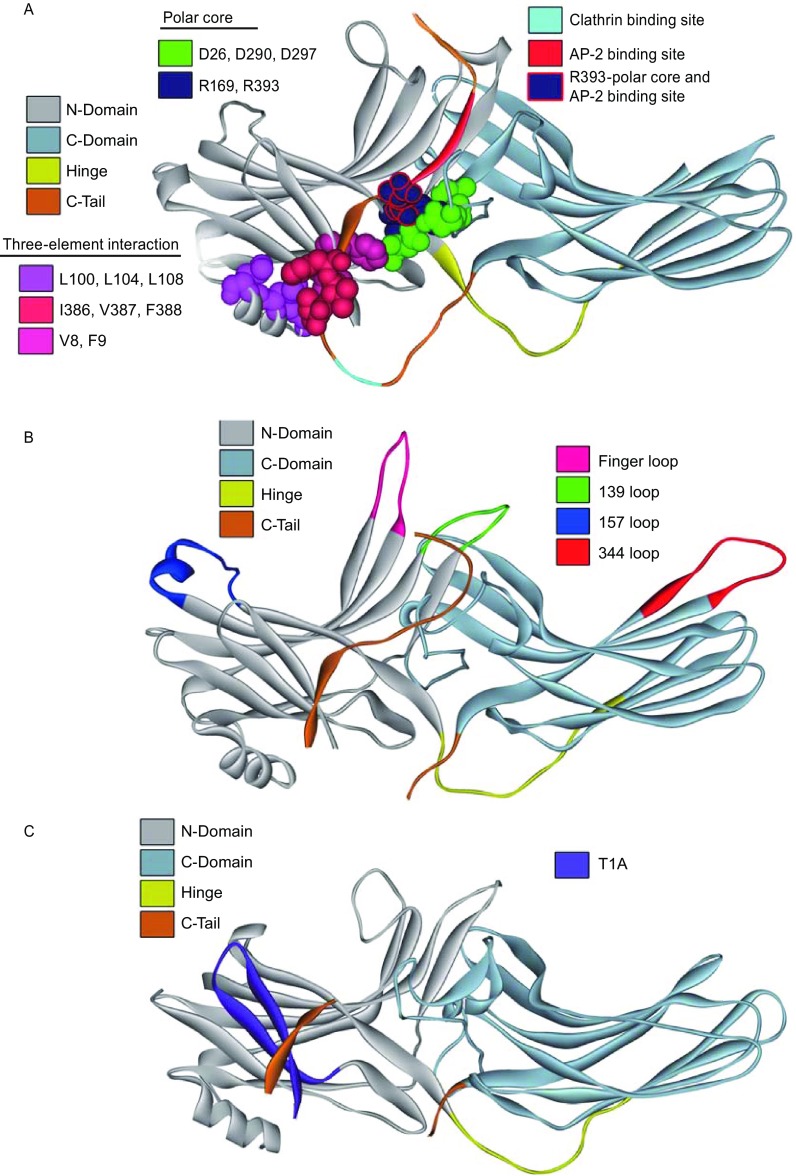
**Localization of key functional elements in arrestins**. (A) Residues of arrestin-2 (PDB ID: 1G4M) involved in critical intra-molecular interactions maintaining the basal conformation are shown as CPK models: in the polar core negatively charged Asp26, Asp290, and Asp297 are colored green, positively charged Arg169 and Arg393 are dark blue; in the three-element interaction the β-strand I residues are salmon, α-helix residues, magenta, and C-tail residues, red. Other structural elements are colored, as follows: inter-domain hinge, yellow; C-tail, brown; main clathrin-binding site, light blue; AP2 binding site, red (note that it includes Arg393, which is also part of the polar core). (B) Parts of arrestin-1 (PDB ID: 1CF1) that move upon receptor binding are colored, as follows: finger loop, salmon; 139-loop, green; 157-loop, dark blue; 344-loop, red. The inter-domain hinge and the C-tail are colored, as in panel A. (C). The position of the JNK3-activating T1A peptide, which includes non-resolved N-terminus and the first two β-strands and the loop between them (magenta), in arrestin-3 (PDB ID: 3P2D) is shown. Note that it is shielded by the C-tail (brown). Inter-domain hinge is shown in yellow

Arrestins are globular proteins found throughout the animal kingdom, with four subtypes present in vertebrates (Gurevich and Gurevich, 2006; Indrischek et al., 2017). Structurally, arrestins are elongated two-domain molecules (Granzin et al., 1998; Hirsch et al., 1999; Han et al., 2001; Milano et al., 2002; Sutton et al., 2005; Zhan et al., 2011) (Fig. 1), with relatively few inter-domain interactions (Fig. 1). Specific binding to active phosphorylated receptor was the first arrestin function discovered (Kuhn et al., 1984). Arrestins suppress G protein activation (Wilden et al., 1986) by direct competition (Wilden, 1995; Krupnick et al., 1997). Crystal structures of active rhodopsin in complex with transducin (visual G protein) peptide (Scheerer et al., 2008), of related G protein-coupled receptor (GPCR)β_2_-adrenergic receptor in complex with heterotrimeric G_s_ protein (Rasmussen et al., 2011), of adenosine A_2A_ receptor with an engineered single-subunit G_s_ protein (Carpenter et al., 2016), and of arrestin-1^1^ complex with rhodopsin (Kang et al., 2015; Zhou et al., 2017) reveal the structural basis for this competition: the C-terminus of G protein α-subunit and central “finger loop” of arrestin-1 (Figs. 1 and 2) occupy the same cavity between the cytoplasmic ends of the α-helices that opens upon activation of rhodopsin (Farrens et al., 1996) and other GPCRs (Rasmussen et al., 2011) (Table 1).

**Table 1:** Crystal structures of arrestin proteins

Arrestin	State	PDB ID	References
Arrestin-1	Basal	1AYR	(Granzin et al., 1998)
Arrestin-1	Basal	1CF1	(Hirsch et al., 1999)
Arrestin-1, p44	Basal	3UGX	(Granzin et al., 2012)
Arrestin-1, p44	Active	4J2Q	(Kim et al., 2013)
Arrestin-1	Active	4ZWJ	(Kang et al., 2015)
Arrestin-1	Active	5W0P	(Zhou et al., 2017)
Arrestin-1-R175E	Intermediate	4ZRG	(Granzin et al., 2015)
Arrestin-2	Basal	1G4R	(Han et al., 2001)
Arrestin-2	Basal	1JSY	(Milano et al., 2002)
Arrestin-2-IP6	Basal	1ZSH	(Milano et al., 2006)
Arrestin-2	Active	4JQI	(Shukla et al., 2013)
Arrestin-3	Basal	3P2D	(Zhan et al., 2011)
Arrestin-3-IP6	Active	5TV1	(Chen et al., 2017)
Arrestin-4	Basal	1SUJ	(Sutton et al., 2005)

Virtually every discovery in the GPCR field was first made in the visual system. The arrestin structure was no exception: the first crystallographic studies revealed three-dimensional structures of visual arrestin-1. Despite their differences in resolution and proposed positions of the N- and C-termini, crystal structures in both studies did not resolve the same elements: the loop between the C-domain and the point of contact that the C-tail makes with the N-domain, as well as distal C-tail (residues 363–404) (Granzin et al., 1998; Hirsch et al., 1999) (Fig. 1). Subsequent structures of arrestin-2 (Han et al., 2001; Milano et al., 2002), arrestin-4 (Sutton et al., 2005), and arrestin-3 (Zhan et al., 2011) also did not reveal these elements. The N-terminus (residues 1–9) is also missing in all structures. Crystal structures indicate disordered elements in several ways. First, some are so flexible that they cannot be even detected in the electron density map: the N-terminus, distal C-terminus, and the connector between the C-domain and the β-strand XX (Figs. 1 and 2). Second, some elements have high B-factors, suggesting that they have alternative positions, but are not disordered enough to become invisible. Third, other elements have distinct conformations in different protomers of crystal oligomer, or in different crystals of the same protein. This applies to many arrestin loops connecting core β-strands in both domains.

The correlation of arrestin structural elements (even those with high structural flexibility) with their functions has been intensively studied. These studies have yielded a significant amount of information on the overall flexibility of these proteins and on the potential roles of such flexibility in arrestin functions. For example, by inserting the six amino-acid motif, C-C-P-G-C-C, into the arrestin-3 (non-visual β-arrestin-2) sequence at sites not directly involved in interactions of this protein with major partners, a set of intramolecular fluorescent arsenical hairpin (FlAsH) bioluminescence resonance energy transfer (BRET) probes (Hoffmann et al., 2005) was created to analyze the effect of various G protein-coupled receptors (GPCRs) on the conformational dynamics and function of this protein (Lee et al., 2016). The analysis using such probes revealed that arrestin-3 structure represents a dynamic conformational ensemble. Different GPCRs are capable of induction of significant structural changes in this protein, generating distinctive arrestin “conformational signatures”, information about which is encoded within the population average arrestin-3 conformation (Lee et al., 2016). It was also shown that in living human cells, the GPCR-arrestin-3 interaction rapidly results in the receptor-specific conformational changes leading to activation of arrestin-3. However, this protein is able to keep receptor-specific conformation and remain active after dissociation from receptors (Nuber et al., 2016). Similarly, different phosphorylated GPCRs were shown to induce distinct structural states of arrestin-2 (β-arrestin-1) required for different specific functions of this protein (Yang et al., 2015). The interaction of visual arrestin (arrestin-1) with light-activated, phosphorylated rhodopsin is accompanied by significant movements of the arrestin loop between β-strands V–VI (“finger loop”) (Fig. 1), suggesting that the ability of arrestin-1 to adopt a high affinity binding state critically depends on the conformational flexibility of this loop (Sommer et al., 2007). A noticeable difference in the conformational flexibility of arrestins-2 and -3 potentially related to their different receptor selectivity has been recently demonstrated using hydrogen/deuterium exchange mass spectrometry (HDX-MS) (Yun et al., 2015). The basal state of arrestin-3 was characterized by higher flexibility of β-strands II, III, and IV (Fig. 1), whereas high dynamics of the middle loop (called “139-loop” in arrestin-1; Figs. 1 and 2) was characteristic for the basal state of arrestin-2. Although both non-visual arrestins became more flexible as a result of their pre-activation, the scale of such pre-activation-induced increase in structural flexibility was noticeably larger in arrestin-2 (Yun et al., 2015). Based on the solution NMR analysis it was concluded that different sets of arrestin-1 regions are utilized for interaction with different functional states of rhodopsin (such as dark-state phosphorylated rhodopsin (P-Rh), phosphorylated opsin (P-opsin), unphosphorylated light-activated rhodopsin (Rh*), and phosphorylated light-activated rhodopsin (P-Rh*)), and that global conformational changes accompany the binding of arrestin-1 to P-Rh* or P-opsin leading to the formation of a dynamic molten globule-like structure. Since activation of arrestin-1 by P-Rh* is achieved via displacement of the long C-tail of arrestin from its polar core by phosphate groups of receptor (Figs. 1 and 2), and arginine 175 located at the polar core serves as the phosphosensor, mutation of this residue to glutamic acid (R175E) produces a pre-active form of arrestin-1 (Granzin et al., 2015). Analysis of the crystal structure of the R175E mutant revealed the presence of noticeable structural differences with the basal state of wild type arrestin-1, most notably several disordered residues in the receptor-binding finger loop and the C-terminus (residues 361–404) (Granzin et al., 2015) (Figs. 1 and 2). Therefore, arrestins in general are characterized by high conformational dynamics, and some of their regions demonstrate high structural flexibility and might be intrinsically disordered.

What does “disorder” as the opposite of “order” mean in relation to protein functionality? The classic paradigm posits that biologically active proteins should have unique rigid 3D structures; therefore, intrinsically disordered proteins (IDPs) can be ignored. However, the reality is more complex. Well before the IDP concept was introduced at the end of 20th century (Wright and Dyson, 1999; Uversky et al., 2000; Dunker et al., 2001), it became clear that protein molecules are inherently flexible, and this flexibility is crucial for their biological activity. In fact, many globular proteins possess marginal conformational stability under physiological conditions, with ΔG_folding_ ranging from −5 to −10 kcal/mol (Privalov and Khechinashvili, 1974; Savage et al., 1993; Ruvinov et al., 1997; Vogl et al., 1997; Giver et al., 1998; Taverna and Goldstein, 2002; Williams et al., 2006). Such marginal stability is related to the protein functionality (Tsou, 1998a, b; Zavodszky et al., 1998), ensuring higher flexibility needed for function-related conformational changes (Artymiuk et al., 1979; Frauenfelder et al., 1979; Wagner and Wuthrich, 1979; Wrba et al., 1990; Varley and Pain, 1991; Daniel et al., 1996; Tang and Dill, 1998; Williams et al., 2006). For example, the internal dynamics of enzymes appears to be linked to the mechanism of catalysis (Agarwal, 2005; Eisenmesser et al., 2005).

Although globular proteins are flexible, some parts are more rigid than others. A protein with several stable structural units can form conformational isomers, determined by its overall flexibility and the locations of the more flexible joints (Ma et al., 1999). Whole proteins or various regions can be ordered or disordered to a different degree (Uversky, 2013). Functional proteins contain differently structured regions, some of which are spontaneously folded (foldons), others fold upon interaction with binding partners (inducible foldons), some are always in semi-folded state (semi-foldons), whereas others do not fold at all (non-foldons) or even need to undergo order-disorder transition to become functional (unfoldons) (Uversky, 2013).

IDPs and hybrid proteins containing ordered domains and IDPRs constitute a very important addition to the more “traditional” set of globular, trans-membrane, and fibrous proteins (Wright and Dyson, 1999; Uversky et al., 2000; Dunker et al., 2001; Uversky and Dunker, 2010; Tompa, 2012; Uversky, 2013; Habchi et al., 2014; van der Lee et al., 2014; Uversky, 2016). The functions of IDPs/IDPRs and ordered proteins/domains are complementary (Dunker et al., 2001; Radivojac et al., 2007). Many IDPs bind multiple partners and act as signaling molecules controlling various aspects of cell behavior (Uversky et al., 2000; Dunker et al., 2001; Iakoucheva et al., 2002; Uversky et al., 2005; Uversky, 2013; Habchi et al., 2014; van der Lee et al., 2014; Uversky, 2015). IDPs/IDPRs are tightly regulated via post-translational modifications (Iakoucheva et al., 2004; Pejaver et al., 2014) and alternative splicing (Romero et al., 2006; Buljan et al., 2013). Dysfunctions of IDPs are associated with human diseases, including amyloidosis, cancer, cardiovascular disorders, and neurodegeneration (Uversky et al., 2008; Uversky et al., 2014).

Arrestins are typical hybrid proteins: they consist of two domains with fairly rigid cores and flexible loops connecting the β-strands, along with mostly disordered C-tail and N-terminus (Fig. 1). Upon arrestin activation, several elements significantly change conformation, some becoming disordered in this state. Here we analyze the connections of structural disorder in arrestins with known functions. The emerging picture is intriguing: structural disorder of arrestin elements appears to be linked to their functionality. The propensity of different parts to be disordered is a surprisingly good predictor of their functional importance.

## Conformational dynamics of arrestins

First, let us explore the basal structure of free arrestins and destabilizing mutations that likely enhance disorder. The idea that the conformations of free and receptor-bound arrestin are dramatically different was first expressed on the basis of high arrestin-1 activation energy (Schleicher et al., 1989), long before any structures were solved. Wild type (WT) arrestin-1 has remarkable selectivity for active phopshorylated rhodopsin (P-Rh*), demonstrating many times lower binding to active unphosphorylated (Rh*) and inactive phosphorylated (P-Rh) forms, and virtually no binding to inactive unphosphorylated rhodopsin (Gurevich and Benovic, 1992, 1993). The mutations that “activate” arrestin-1, facilitating its binding to non-preferred forms of rhodopsin, fall into four categories: 1) C-terminal deletions (Gurevich and Benovic, 1992) and alanine substitutions of hydrophobic residues in the C-tail (Gurevich, 1998), which eliminate the C-tail or release it from its basal position; 2) short N-terminal deletions (Gurevich and Benovic, 1993; Gurevich et al., 1994); 3) charge elimination/reversal mutations in the polar core (Hirsch et al., 1999) (Fig. 2A), particularly Arg-175 (Gurevich and Benovic, 1995, 1997) in arrestin-1, or homologous Arg-169 in arrestin-2 (Kovoor et al., 1999; Pan et al., 2003) and Arg-170 in arrestin-3 (Celver et al., 2002); 4) point mutations on the receptor-binding surface (Hanson and Gurevich, 2006; Vishnivetskiy et al., 2013) and small deletions of the “139-loop” in the central crest of the molecule (Figs. 1 and 2B) (Vishnivetskiy et al., 2013). Curiously, the first three categories of mutations affect residues that, according to the crystal structure of the complex (Kang et al., 2015; Zhou et al., 2017), are not directly involved in receptor binding.

Crystal structure of free arrestin-1 revealed two intra-molecular interactions that serve as “clasps” holding the molecule in the basal state (Granzin et al., 1998; Hirsch et al., 1999) (Fig. 2A). The first three types of activating mutations disrupt these clasps. One of these clasps, the polar core, is an arrangement of five interacting charged residues between the two arrestin domains (Fig. 2A). These charges are virtually solvent-excluded (Hirsch et al., 1999), which is very unusual for a soluble protein, where charged side chains are normally solvent-exposed. The other clasp is the three-element interaction of β-strand I and α-helix in the N-domain, and β-strand XX in the C-tail (Hirsch et al., 1999) (Figs. 1 and 2A). The C-tail binds to the N-domain via hydrophobic residues, replacement of which with alanines “activates” arrestin-1 (Gurevich, 1998). Thus, for high-affinity binding, active phospho-receptor needs to disrupt key interactions that support basal conformation (Gurevich and Gurevich, 2004), thereby making arrestin more flexible. Receptor-attached phosphates destabilize both the polar core (Vishnivetskiy et al., 1999) and three-element interaction (Vishnivetskiy et al., 2000), enabling transition into high-affinity receptor-binding state. Scanning mutagenesis of the whole arrestin-1 molecule (Ostermaier et al., 2014) confirmed earlier findings regarding the role of the polar core and three-element interaction in maintaining the basal arrestin conformation, as well as the role of Arg18 and Lys20 in phosphate binding (Hanson and Gurevich, 2006). In addition, this comprehensive study revealed the role of Arg29 in phosphate binding that was not explored before (Ostermaier et al., 2014). It also confirmed the role of the finger loop (Fig. 1) in receptor binding proposed earlier (Hanson et al., 2006b; Vishnivetskiy et al., 2011) and suggested that the lariat loop containing two of the three negative charges in the polar core (Fig. 2A) also engages the receptor (Ostermaier et al., 2014). The most unexpected was the finding that the distal tip of the C-domain (part of 344 loop in Fig. 2B) likely engages the membrane (Ostermaier et al., 2014), which was subsequently confirmed by fluorescence quenching experiments (Lally et al., 2017).

Several methods, such as hydrogen/deuterium exchange (Carter et al., 2005), measurements of spin label mobility in free and receptor-bound arrestin-1 by EPR (Hanson et al., 2006b), and NMR of isotopically labeled arrestin-1 (Zhuang et al., 2010; Zhuang et al., 2013a), indicate that in both free and receptor-bound states arrestin-1 is flexible, likely exploring a wide range of conformations. This applies to disordered elements that were not resolved in crystal and parts that clearly show up in 3D structures. Several loops demonstrate conformational variability in different protomers within crystallographic tetramer of arrestin-1 (Hirsch et al., 1999), or in different crystal forms of arrestin-2 (Han et al., 2001). Structural variability within the same protein is as great as the variations between different subtypes, indicating that it reflects flexibility of these regions, rather than their subtype-specific conformations. Although arrestins appear to assume another distinct conformation upon binding to microtubules (Hanson et al., 2006a; Hanson et al., 2007), due to the lack of structural information about this state we will limit the discussion to free and GPCR-bound forms.

**Figure 3:**
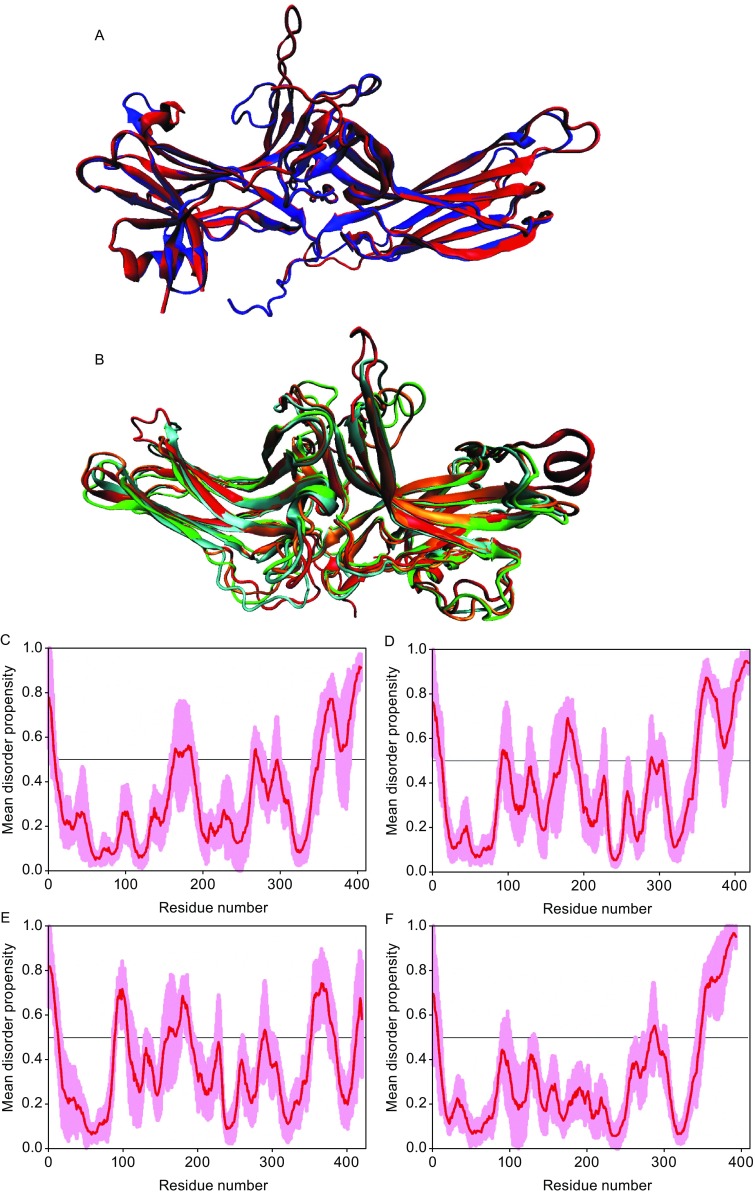
**Structural disorder in arrestin proteins**. (A) Structural alignment of PDB entries 1AYR (blue ribbon) and 1CF1 (red ribbon) corresponding to the crystal structures of visual arrestin-1 from bovine rod photoreceptors. (B) Multiple structure alignments for bovine arrestin-1 (PDB ID: 1CF1, red ribbon), arrestin-2 (PDB ID: 1G4M, green ribbon), arrestin-3 (PDB ID: 3P2D, cyan ribbon), and arrestin-4 (PDB ID: 1SUJ, orange ribbon). Structures were generated using VMD 1.9.2 (Humphrey et al., 1996). Multiple structure alignment was performed using the MultProt (http://bioinfo3d.cs.tau.ac.il/MultiProt/) (Shatsky et al., 2004). (C–F) Multiparametric computational analysis of the intrinsic disorder predisposition of bovine arrestin-1 (UniProt ID: P08168) (C), arrestin-2 (UniProt ID: P17870) (D) arrestin-3 (UniProt ID: P32120) (E), and arrestin-4 (UniProt ID: Q9PTE7) (F). In each plot, red line shows the mean disorder propensity calculated by averaging disorder profiles generated by six common disorder predictors: PONDR^®^ VLXT (Dunker et al., 2001), PONDR^®^ VSL2 (Peng et al., 2005), PONDR^®^ VL3 (Peng et al., 2006), PONDR^®^ FIT (Xue et al., 2010), and IUPred_short and IUPred_long (Dosztanyi et al., 2005). Light pink shadow around the mean disorder score shows distribution of standard deviations. Predicted intrinsic disorder scores above 0.5 are considered to correspond to the disordered regions, whereas regions with the disorder scores between 0.2 and 0.5 are considered flexible

To illustrate the range of structural flexibility in the visual arrestin-1, Fig. 3A shows two structures of this protein (PDB IDs: 1AYR and 1CF1) with several loops demonstrating noticeable structural divergence. Whereas in the structures of arrestin-1 (Hirsch et al., 1999), arrestin-2 (Han et al., 2001), and arrestin-3 (Sutton et al., 2005) some of these loops participate in contacts with other protomers, their structural differences do not seem to be explained solely by lattice contacts: the first β-strand on the concave face of the N-domain and the first β-strand on the convex face of the C-domain are engaged by the sister protomers in all arrestin crystals (Zhan et al., 2011), yet their conformation is identical in all protomers. Fig. 3B provides structural alignment of bovine arrestin-1 (1CF1), arrestin-2 (1G4M), arrestin-3 (3P2D), and Salamander arrestin-4 (1SUJ) and shows that the greatest structural variability is observed in the loop regions. Fig. 3C–E show disorder profiles generated for these arrestins by several common disorder predictors. Both N- and C-termini are predicted to be disordered (disorder scores exceed the threshold of 0.5). Several internal regions with disorder scores between 0.2 and 0.5 are expected to have high conformational flexibility. Sequence analysis suggests that arrestin-3 is the most disordered subtype (Fig. 3). Indeed, it is the most promiscuous non-visual arrestin with the lowest preference for phosphorylated over unphosphorylated receptor (Celver et al., 2002; Zhan et al., 2011), the lowest receptor subtype selectivity (Barak et al., 1997; Zhan et al., 2011), and higher affinity for many non-receptor partners (Goodman et al., 1996; Ahmed et al., 2011). Molecular dynamics simulations suggest that the flexibility of wild type arrestin-3 approaches that of the pre-activated R175E mutant of bovine arrestin-1 (Sensoy et al., 2016). The crystal structures confirm conformational flexibility of elements predicted to be disordered by sequence analysis: N- and C-termini are not resolved in the crystal structures, loops in the central crest of the molecule and at the tips of both domains are identifiable in the electron density maps and therefore resolved in most cases, but assume distinct conformations in different protomers in crystal oligomers and in different crystal forms of the same protein.

## Disordered elements of arrestins contain key binding sites for clathrin and AP2

The binding to active phosphorylated GPCRs was long considered the only function of arrestins. Clathrin (Goodman et al., 1996) and clathrin adaptor AP2 (Laporte et al., 1999) were the first discovered non-receptor binding partners of arrestins. Clathrin binds the loop between β-strands XIX and XX, whereas AP2 interacts with the distal C-tail (Fig. 2A), both disordered elements of arrestin (Fig. 1) not resolved in crystal structures. Longer isoform of arrestin-2 contains an additional clathrin binding site also localized in the disordered part (Kang et al., 2009). Interestingly, when these arrestin elements are co-crystallized with clathrin, both demonstrate highly ordered structures (ter Haar et al., 2000; Kang et al., 2009), in line with the idea of coupled binding and folding (Shoemaker et al., 2000; Sugase et al., 2007).

Disorder does not mean that these elements are equally accessible in free and receptor-bound arrestins. The release of the C-tail in all arrestins is induced by receptor binding (Palczewski et al., 1991; Hanson et al., 2006b; Vishnivetskiy et al., 2010), and poly-anions heparin (Palczewski et al., 1991; Vishnivetskiy et al., 2002; Zhuang et al., 2010), IP6 (Zhuang et al., 2010; Chen et al., 2017), and multi-phosphorylated C-terminus of rhodopsin (Puig et al., 1995) or vasopressin V2 receptor (Xiao et al., 2004; Nobles et al., 2007). While free arrestin-2 and arrestin-3 bind clathrin (Goodman et al., 1996), the affinity of both arrestins is greatly increased by the release of the C-tail (Xiao et al., 2004; Nobles et al., 2007). Activating mutations destabilizing inter-domain polar core significantly enhance clathrin and AP2 binding (Milano et al., 2002), likely by increasing overall flexibility of the molecule and the probability of the C-tail release in free arrestins. The C-tail release was observed upon a small deletion in 139-loop (Kim et al., 2012), which is also an activating mutation increasing arrestin binding to non-preferred forms of rhodopsin (Vishnivetskiy et al., 2013). Thus, while the range of conformations sampled by the arrestin elements that bind clathrin and AP2 in the basal state is wide enough to prevent their visibility in crystal, it shifts upon arrestin binding to receptors, making the C-tail even more flexible.

However, the order (or lack thereof) is not the only factor affecting the interactions of the C-tail of non-visual arrestins with the trafficking machinery. It has been reported that phosphorylation of arrestin-2 at Ser-412 by ERK1/2 inhibits its interactions with clathrin (and possibly clathrin adaptor AP2, that binds the distal part of the arrestin C-tail (Laporte et al., 1999)) (Fig. 2A) and arrestin-dependent internalization of βAR (Lin et al., 1997). Similar consequences of arrestin-3 phosphorylation at Thr-383 by casein kinase II were also described (Kim et al., 2002; Lin et al., 2002). In both cases receptor binding and consequent release of the C-tail was shown to facilitate dephosphorylation of non-visual arrestins, which enhances clathrin binding just when it is needed to recruit the arrestin-receptor complexes to the coated pit (Lin et al., 1997, 2002; Kim et al., 2002). It is unlikely that phosphorylation/dephosphorylation of these inherently disordered parts of the arrestin C-tail affects their secondary structure. Thus, the presence or absence of phosphates likely directly regulates the interactions of the arrestin C-tail with components of the internalization machinery of the coated pit.

An unexpected effect of PKC phosphorylation of arrestin-2 (β-arrestin1) has been described in the process of T cell activation (Fernández-Arenas et al., 2014). Sustained signaling requires constant supply of antigen-binding T cell receptors to the immunological synapse. To achieve this, T cell needs to internalize peripheral T cell receptors not engaged by the antigen and then deliver them to the immunological synapse, where they can bind the antigen and become activated. Arrestin-2 appears to mediate this process upon its phosphorylation at Ser-163, localized in the cavity of the N-domain (Fig. 1), where the GPCR-attached phosphates bind (Shukla et al., 2013; Zhuo et al., 2014). This phosphorylation apparently activates arrestin-2, with the phosphate attached by PKC (which is activated by T cell receptors) to arrestin itself acting as its activator in lieu of receptor-attached phosphates (Gurevich and Gurevich, 2014). While exact mechanism of triggering the arrestin-2 phosphate sensor by the phosphate attached to Ser-163 was not elucidated, triggering of the phosphate sensor by other means, including polar core mutations and bound polyanions, has been shown to release the C-tail of arrestins (Kim et al., 2002; Zhuang et al., 2010) and increase the affinity of non-visual arrestin subtypes for the components of internalization machinery (Kim et al., 2002; Xiao et al., 2004).

**Figure 4:**
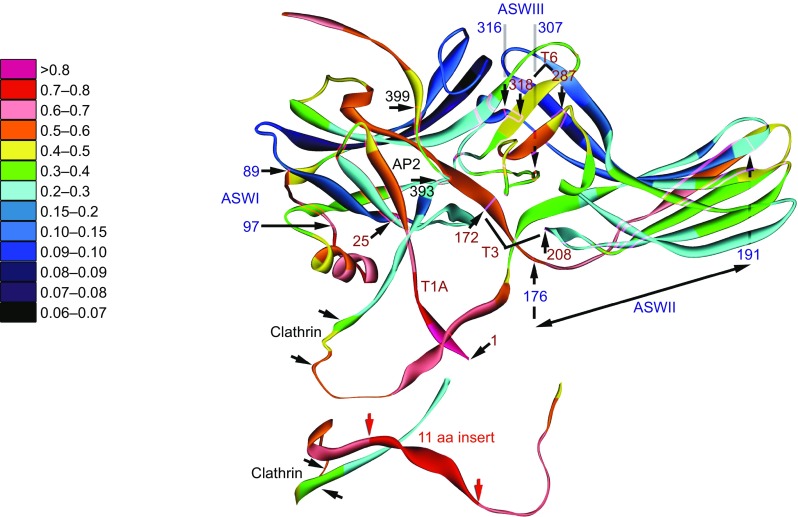
**Functional implications of structural disorder**. The structure of the basal state of arrestin-3 (PDB ID: 3P2D (Zhan et al., 2011)) is colored according to the propensity for structural disorder (color-code shown on the left). The C-terminus of the long splice variant of arrestin-3 with an 11-residue insert (as compared to the short splice variant (Sterne-Marr et al., 1993)) is shown below. Known functional elements are indicated on the structure: T1A peptide that facilitates the activation of JNK3 in cells (Zhan et al., 2016), residues 1–25; clathrin-binding element (residues 373–377 in the short splice variant), clathrin adaptor AP2 binding site (residues 393–399 in the short splice variant); JNK3-binding peptides T3 (residues 172–208) and T6 (residues 287–318) (Zhan et al., 2014); as well as arrestin switches that significantly change conformation upon activation (Chen et al., 2017): ASwI (residues 89–97), ASwII (residues 176–191), and ASwIII (residues 307–316). Note that JNK3-binding peptides T3 and T6 include ASwII and ASwIII, respectively. Also note that known functional elements are localized in regions of predicted high or moderate disorder. The 11-residue insert in the long splice variant (Sterne-Marr et al., 1993) has very high disorder score. Alternative splicing of arrestin-3 is conserved in mammalian evolution (Gurevich and Gurevich, 2006; Indrischek et al., 2017), suggesting that this insert is likely functionally important, e.g., binds an unknown partner that the short variant does not bind

Molecular recognition of IDPs and IDPRs is associated with their ability to undergo a disorder-to-order transition upon binding to their partners (Dunker and Obradovic, 2001; Dyson and Wright, 2002; Uversky et al., 2005). There are elements, known as molecular recognition features (MoRFs), located within longer IDPRs, that are disordered in their unbound state and fold upon interaction with specific partners (Oldfield et al., 2005), mediating IDP/IDPR binding via disorder-to-order-transition-based recognition involved in many biological processes (Disfani et al., 2012). Often, in corresponding disorder profiles, MoRFs are indicated as “dips”; i.e., regions with locally increased order propensity within long IDPRs. Potential MoRFs in arrestins (the regions spanning residues 372–383 in arrestin-1, residues 143–151, 165–170, 343–351, and 375–397 in arrestin-2, residues 143–153 and 167–172 in arrestin-3, and residues 341–348 and 364–371 in arrestin-4) were identified by the ANCHOR algorithm (Dosztanyi et al., 2009). Figure 4 maps known functional elements and arrestin switches (ASw, regions that significantly change conformation upon activation) on arrestin-3 structure. These functional elements include T1A peptide (residues 1–25) needed for the JNK3 activation (see also Fig. 2C) (Zhan et al., 2016), JNK3-binding peptides T3 (residues 172–208) and T6 (residues 287–318) (Zhan et al., 2014), ASwI (residues 89–97), ASwII (residues 176–191), and ASwIII (residues 307–316) (Chen et al., 2017), clathrin-binding element (residues 373–377), and clathrin adaptor AP2 binding site (residues 393–399). Importantly, ASwII and ASwIII are parts or the JNK3-binding peptides T3 and T6, respectively. Also, JNK3-binding peptide T3 and ASwII are located in close proximity of one of the MoRFs (residues 167–172). In addition to two real MoRFs, ANCHOR profile for arrestin-3 contains 9 filtered potential disorder-based regions (which are either short regions with length below 6 residues or regions with an average disorder score below 0.1), residues 80–82 (located in close proximity of ASwI), 116–120, 208–209 (overlaps with C-terminal region of the JNK3-binding peptide T3), 238–247, 271–274, 317–326 (located next to ASwIII), 342, 399 (located within clathrin adaptor AP2 binding site), and 402. Therefore, the comparison of Figs. 3 and 4 shows that functional significance of several MoRFs is already established, whereas the role of other disorder-based binding sites in arrestin interactions with various proteins needs to be tested experimentally.

## Can the “ordered” part of arrestin become “disordered”?

The ~350-residue core of all arestins, from residue ~10 to ~360, is relatively well ordered in all crystal structures of the basal state, with the exception of loops connecting β-strands. C-terminal truncations by alternative splicing (Smith et al., 1994; Pulvermuller et al., 1997) or mutagenesis (Gurevich and Benovic, 1992, 1993) significantly reduce thermal stability of arrestin-1, implying increased flexibility of the remaining part, which lowers energy barrier for the receptor interaction (Pulvermuller et al., 1997; Ahmed et al., 2011) and significantly enhances the arrestin binding to non-preferred forms of GPCRs (Gurevich, 1998; Celver et al., 2002), supporting the idea that less ordered proteins are more inclined to bind their partners. A virtually perfect correlation between increased binding of arrestin-1 mutants to non-preferred forms of rhodopsin and the reduction of their thermal stability was established (Vishnivetskiy et al., 2013). Interestingly, the effects of the C-terminal deletions are not limited to GPCR interactions, facilitating the binding of some non-receptor partners.

A recent study illustrates how nature uses this phenomenon as a gain-of-function change. A common feature of apoptotic cell death is a massive activation of specialized proteases, caspases, that cleave numerous structural and signaling proteins (Lüthi and Martin, 2007), yielding characteristic morphological and biochemical changes. The cleavage of arrestin-2 by caspases at Asp-380 generates a truncated form lacking the C-tail, arrestin-2_1–380_ (Kook et al., 2014). Full-length (FL) arrestin-2 is evenly distributed in the cell (Barak et al., 1997; Song et al., 2006), whereas arrestin-2_1–380_ is recruited to mitochondria, even though it does not have recognizable mitochondrial localization signal. In contrast to FL protein, arrestin-2_1–380_ avidly binds another product of caspase activity, truncated Bid (tBid). Although tBid localizes to mitochondria, arrestin-2_1–380_ moves to this compartment independently, because its mitochondrial localization does not require the presence of tBid or Bid (Kook et al., 2014). Thus, the C-terminal truncation of arrestin-2 confers two new functions: the ability to bind mitochondria and tBid. Both are equally important for the role of arrestin-2_1–380_ in apoptosis, where it enhances tBid-induced cytochrome *c* release from mitochondria, which is the point of no return in the apoptotic cell death (Danial and Korsmeyer, 2004). Apparently, the removal of the C-tail by caspases promotes arrestin-2 interactions with tBid and resident mitochondrial protein(s) by increasing its flexibility, helping arrestin-2_1–380_ fit partners that the more rigid FL protein does not bind. Similarly, C-terminal deletions or C-tail detachment enhance the binding of all arrestin subtypes to microtubules (Hanson et al., 2007).

However, increased flexibility of the core does not indiscriminately enhance arrestin interactions with all possible partners. The C-tail of all arrestins can be forcibly detached by triple alanine substitution of the hydrophobic residues that anchor it to the N-domain (3A) (Gurevich, 1998; Celver et al., 2002; Pan et al., 2003; Song et al., 2009b) (Fig. 2A). These 3A mutations increase the flexibility of the molecule, as revealed by faster H/D exchange (Carter et al., 2005), and reduce activation energy for receptor binding (Gurevich et al., 2011), suggesting that these mutants are in conformation intermediate between basal and receptor-bound. The opposite conformational bias is conferred by seven-residue deletion in the inter-domain hinge (Δ7) (Figs. 1 and 2) that strongly inhibits receptor binding (Vishnivetskiy et al., 2002; Hanson et al., 2007).

Interestingly, the binding of two E3 ubiquitin ligases, Mdm2 and parkin, is inhibited by 3A and enhanced by Δ7 mutations (Song et al., 2006; Ahmed et al., 2011). These data do not necessarily demonstrate that these partners prefer “ordered” arrestins over “disordered”. It has been shown that Δ7 mutation also results in the C-tail release (Hanson et al., 2006a), suggesting that it produces a different flavor of disorder, conducive to the binding of Mdm2 and parkin. However, these flavors must have some similarities: the binding of ERK1/2 is facilitated by both 3A and Δ7 mutations in arrestin-2 and -3 (Coffa et al., 2011a). Thus, different mechanisms of destabilization of the basal arrestin structure appear to increase its flexibility in distinct partially overlapping ways, enhancing the binding of different, or sometimes the same, partners.

## Flexibility of receptor-bound arrestins

The arrestin-receptor complexes initiate several signaling cascades that free arrestins do not (Peterson and Luttrell, 2017). For example, ERK1/2 binds free arrestin with low affinity (Luttrell et al., 2001; Coffa et al., 2011a), whereas this interaction is greatly enhanced by the arrestin recruitment to the receptor (Luttrell et al., 2001). The arrestin-dependent ERK1/2 activation occurs only upon GPCR stimulation (Luttrell et al., 2001; Coffa et al., 2011b). From structural perspective, the simplest explanation is that receptor-bound arrestin presents a different “face” to the world, where elements engaged by particular partners, such as ERK1/2, become more accessible or better aligned (Gurevich and Gurevich, 2003). However, for a long time the release of the C-tail remained the only conformational rearrangement in arrestins directly ascribed to the receptor binding. Recent study using pulse EPR technique DEER for intra-molecular distance measurements in free and P-Rh*-bound arrestin-1 detected additional rearrangements. These included the movement of the finger loop on the receptor-binding surface (Figs. 1 and 2B) in the direction of the incoming receptor, a dramatic repositioning of the neighboring 139-loop towards the N-domain and to the side, and movements of two loops containing residues 344 and 157 at the tips of the two arrestin domains (Fig. 2B) towards and away from the receptor, respectively (Kim et al., 2012). Interestingly, the 344 loop in the stretch with elevated disorder potential in all arrestins (Fig. 3C–F) directly engages membrane lipids (Lally et al., 2017), consistent with its position in the arrestin-1-rhodopsin complex (Kang et al., 2015).

Earlier findings that deletions in the inter-domain hinge impede the receptor binding (Vishnivetskiy et al., 2002; Hanson et al., 2007) suggested that long hinge is needed for the two arrestin domains to “grab” the receptor like a pincer (Gurevich and Gurevich, 2004). However, DEER studies of arrestin-1 (Kim et al., 2012) and both non-visual subtypes (Zhuo et al., 2014) ruled out such “clam-like” movement of the domains. In an alternative model, the receptor binding is enabled by the two domains rotating relative to each other by ~20° (Modzelewska et al., 2006). Two crystal structures of presumably “active” arrestin-1 and arrestin-2 are consistent with this model. Pre-activated arrestin-2 with C-terminal deletion (Kovoor et al., 1999) was co-crystallized with multi-phosphorylated C-terminus of vasopressin V2 receptor and conformationally selective antibody fragment (Shukla et al., 2013). This structure revealed a large movement of the 139-loop homologue (called “middle loop” in this study), similar to that detected in arrestin-1 by DEER (Kim et al., 2012), rearrangement of the finger loop, and the rotation of N- and C-domains by ~20^o^ (Shukla et al., 2013). The short splice variant of arrestin-1 lacking the C-tail, p44, expressed in some mammalian species, was also shown to be “pre-activated” and bind unphosphorylated Rh* (Pulvermuller et al., 1997). In one crystal structure, this protein looked similar to FL arrestin-1 (Granzin et al., 2012), but the second structure revealed a very different conformation, with characteristic rearrangement of the finger loop, movement of the 139-loop, and ~21° rotation of the two domains (Kim et al., 2013).

These studies have obvious caveats. Both DEER (Kim et al., 2012) and NMR (Zhuang et al., 2013a) were done with arrestin-1 bound to a bona fide receptor, purified rhodopsin, but neither method yields atomic resolution. In higher resolution crystal structures, “active” arrestin-1 (Kim et al., 2013) and arrestin-2 (Shukla et al., 2013) were not bound to a receptor. The recent crystal structure of the arrestin-1-rhodopsin complex, the only structure of the GPCR-bound arrestin so far, solved using innovative method suitable for very small crystals, femtosecond X-ray laser, revealed similar movements of flexible loops and the twisting of the two arrestin-1 domains (Kang et al., 2015; Zhou et al., 2017).

GPCRs are very dynamic proteins (Manglik and Kobilka, 2014; Manglik et al., 2015), which makes them challenging targets for X-ray crystallography. Virtually all structures were solved using heavily engineered receptors, where some elements were deleted and/or replaced with small soluble proteins that provide crystal contacts. The arestin-1-rhodopsin complex was no exception. First, a fusion of human rhodopsin with T4 lysozyme was used. Second, this rhodopsin carried two mutations, M257Y and E113Q, making it fully active even in the absence of retinal, and an additional disulfide bridge between residues 2 and 282 that were replaced with cysteines, which is widely used for rhodopsin stabilization (Standfuss et al., 2007). Third, rhodopsin was not phosphorylated to the level required for tight wild type arrestin-1 binding (Mendez et al., 2000; Vishnivetskiy et al., 2007). Thus, pre-activated mouse arrestin-1-3A mutant that binds Rh* with high affinity was used (Song et al., 2009b; Vishnivetskiy et al., 2013). Finally, although individually expressed rhodopsin and arrestin-1 mutants were able to bind each other, a complex stable enough for crystallization was obtained only when both were expressed as a fusion protein. However, the arrangement of arrestin-1 and rhodopsin in the structure of the complex that was expressed and crystallized as a fusion protein, was confirmed using separately expressed and purified arrestin-1 and rhodopsin by several methods: a) changes in the rate of H/D exchange upon binding; b) disulfide cross-linking between residues in arrestin-1 and rhodopsin predicted to be within range by the crystal structure, and lack of cross-linking between residues predicted to be too far apart; and c) measurements of distances between selected positions in the two proteins using DEER (Kang et al., 2015; Zhou et al., 2017).

Well-diffracting crystals form only when protein conformations are fixed, either naturally, or artificially, by crystallization conditions and/or crystal packing forces. In this crystal, all four arrestin-rhodopsin complexes in the asymmetric unit look very similar (Kang et al., 2015; Zhou et al., 2017), strongly suggesting biological relevance of this structure. In the arrestin-rhodopsin complex three distances were measured by DEER: from rhodopsin residue 74 to arrestin-1 residues 61, 140, and 241 (Kang et al., 2015); four distances were measured to confirm the position of the rhodopsin C-terminus relative to the N-domain of arrestin-1: from rhodopsin positions 335, 337, and 342 to residue 107 in arrestin-1, and from 342 in rhodopsin to 106 in arrestin-1 (Zhou et al., 2017). It is remarkable that the most probable distance in each case matched the crystal structure. However, it is equally remarkable that in every case, multiple additional distances were detected, clearly indicating that the complex explores fairly wide conformational space (Kang et al., 2015; Zhou et al., 2017). This notion is supported by the finding that the distributions of intra-molecular distances measured by DEER in free arrestin-1 are wide, and remain wide upon rhodopsin binding (Kim et al., 2012). The same is true for intra-molecular distances in free and receptor-bound arrestin-2 and -3 (Zhuo et al., 2014). NMR data for ^13^C-^15^N-labeled arrestin-1 also suggest its high flexibility in rhodopsin-bound form (Zhuang et al., 2013a). Thus, all evidence indicates that receptor-bound arrestins, which organize a variety of signaling complexes, are very flexible. This appears to be necessary for their functional versatility.

Arrestin-dependent activation of JNKs illustrates one mechanism whereby flexibility translates into function. Out of the four vertebrate arrestins, only arrestin-3 activates JNKs (McDonald et al., 2000; Song et al., 2009a, 2011). Highly homologous arrestin-2 (Attramadal et al., 1992; Sterne-Marr et al., 1993) also binds kinases in the ASK1-MKK4-JNK3 cascade, but does not activate JNKs (Song et al., 2009a, 2011). Although JNK3 and MKK4 binding was discovered first (McDonald et al., 2000), subsequent studies showed that arrestin-3 also binds MKK7 (Zhan et al., 2013) needed for full activation of JNKs (Lawler et al., 1998), as well as ubiquitously expressed JNK1 and JNK2 (Kook et al., 2013). Therefore, the mechanism described below applies to arrestin-3 scaffolding of all JNK activation cascades.

JNK3 engages three arrestin-3 elements (Fig. 4), with the N-terminal 25 residues (T1A peptide) being the most potent binding site (Zhan et al., 2014). This peptide binds all kinases in the pathway and facilitates JNK activation *in vitro* and in cells (Zhan et al., 2016). The concentration dependence of the scaffold effect is usually bell-shaped (Levchenko et al., 2004): low scaffold concentrations facilitate productive interactions, which are suppressed when there is more scaffold molecules than the components of the cascade. The optimal concentration of the scaffold yielding the highest reaction rate depends on scaffold affinities for the proteins it brings together (Zhan et al., 2013). Interestingly, optimal concentrations of T1A peptide for scaffolding of MKK4-JNK3 and MKK7-JNK3 modules are 10 times lower than those of FL arrestin-3 (Zhan et al., 2016). Arrestin-3 structure suggests an explanation for this. Even though free arrestin-3 acts as a scaffold (Song et al., 2009a; Breitman et al., 2012), receptor binding enhances this action (McDonald et al., 2000). In the basal state of arrestin-3, T1A peptide, which includes the N-terminal extension not resolved in crystal and β-strands I and II (Zhan et al., 2014) (Figs. 1 and 4), is partially shielded by the C-tail (Fig. 2C), which is released upon receptor binding (Zhuo et al., 2014), ensuring better exposure of this element. This explains higher effectiveness of the receptor-bound arrestin-3 as JNK3 activator (McDonald et al., 2000), as well as higher apparent affinity of T1A for JNK3, MKK4, and MKK7 (Zhan et al., 2016). Furthermore, expressed as a YFP-fusion, T1A peptide is likely fully exposed and flexible, activating JNK3 more efficiently than full-length arrestin-3 (Zhan et al., 2016). Thus, enhanced flexibility of a protein element facilitates its function.

## Does disorder potential have predictive value?

It is instructive to compare disorder potential of arrestin elements (Fig. 3C–F) with available functional data (Fig. 4). The N-terminus and the C-tail, with high predicted disorder in all arrestins (Fig. 3C–F), are biologically important. The first 25 residues of arrestin-3 (Figs. 2C and 4) are the highest affinity binding site for JNK3 (Zhan et al., 2014), bind all the upstream kinases in JNK3 activation cascade, and serve as a functional scaffold in cells (Zhan et al., 2016). The part of the C-terminus of both arrestin-2 and -3 between the C-domain and β-strand XX (Fig. 1), which is disordered and never resolved in crystal structures, contains both the major (Goodman et al., 1996) and minor (in arrestin-2) (Kang et al., 2009) clathrin-binding sites (Fig. 2A). The distal C-tail (also never resolved in crystal structures) binds clathrin adaptor AP2 (Laporte et al., 1999; Milano et al., 2002; Moaven et al., 2013) (Fig. 2A). Upon receptor binding, when the interactions with clathrin and AP2 come into play, the C-tail of arrestins is released and becomes even more disordered. The comparison of the structure of bovine arrestin-3 activated by IP6 (Chen et al., 2017) with the structures of arrestin-2 activated by receptor phospho-peptide (Shukla et al., 2013), pre-activated arrestin-1 splice variant p44 (Kim et al., 2013), and arrestin-1 in complex with rhodopsin (Kang et al., 2015; Zhou et al., 2017) identified three regions that invariably assume different conformations in basal and active arrestins, termed arrestin switch (ASw) regions (Chen et al., 2017). These include ASwI (one of the long “legs” connecting the α-helix to the body of the N-domain, residues 89–97 in bovine arrestin-3), ASwII (inter-domain hinge and the first β-strand in the C-domain, residues 176–191), and ASwIII (the extension of the lariat loop supplying two of the three negative charges of the polar core, residues 307–316) (Fig. 4). Switches likely serve as docking sites for the non-receptor partners that discriminate between active and inactive arrestins (Chen et al., 2017). Interestingly, all these elements are predicted to have high disorder potential in both non-visual subtypes (Figs. 3D, 3E, and 4) implicated in arrestin-mediated signaling, and appear to be more ordered in visual arrestin-1 and -4 (Fig. 3C and 3F), which lack these functions. Importantly, two other regions implicated in JNK3 binding, peptides T3 (residues 172–208) and T6 (residues 287–318) (Zhan et al., 2014) contain ASwII and ASwIII, respectively (Chen et al., 2017) (Fig. 4). While other protein partners that engage different switch regions still await identification, there is a provocative tendency for the elements with the highest disorder potential to mediate arrestin interactions with other proteins.

## Conclusions

Biologically, the most remarkable characteristic of arrestins is their ability to bind hundreds of different GPCRs and numerous non-receptor partners. Arrestins exist in at least three distinct conformations: free, receptor-bound, and microtubule-bound. Structurally, an important feature of arrestins is high flexibility in every state. X-ray crystallography and biophysical studies clearly show that in both free and receptor-bound state arrestins explore a wide range of conformations. Conceivably, the conformational spaces covered by free, receptor- and microtubule-bound arrestins partially overlap. This flexibility likely underlies remarkable functional versatility of arrestins and represents the structural basis for the ability of arrestins to scaffold signaling pathways. The functionality of free arrestins and their separated elements paves the way for the use of arrestin-based molecular tools to manipulate cellular signaling for experimental and, potentially, therapeutic purposes. The correlation of functional capabilities with high probability of disorder suggests that in search of arrestin elements engaging signaling proteins the field needs to pay closer attention to more disordered parts.
